# The elicitation of patient and physician preferences for calculating consumer-based composite measures on hospital report cards: results of two discrete choice experiments

**DOI:** 10.1007/s10198-023-01650-2

**Published:** 2023-12-15

**Authors:** Martin Emmert, Stefan Rohrbacher, Florian Meier, Laura Heppe, Cordula Drach, Anja Schindler, Uwe Sander, Christiane Patzelt, Cornelia Frömke, Oliver Schöffski, Michael Lauerer

**Affiliations:** 1 Faculty of Law, Business and Economics, Institute for Healthcare Management and Health Sciences, University of Bayreuth, Prieserstraße 2, 95444 Bayreuth, Germany; 2 Department of Management and Economics, SRH Wilhelm Löhe University of Applied Sciences, 90763 Fürth, Germany; 3 School of Business and Economics, Chair of Health Care Management, Friedrich-Alexander-University of Erlangen-Nuremberg, Lange Gasse 20, 90403 Nuremberg, Germany; 4 Department of Information and Communication, Faculty for Media, Information and Design, University of Applied Sciences and Arts, Hannover, Germany

**Keywords:** Public reporting, Hospital choice, Discrete choice experiment, Composite measures, Hospital report cards, I12

## Abstract

**Purpose:**

The calculation of aggregated composite measures is a widely used strategy to reduce the amount of data on hospital report cards. Therefore, this study aims to elicit and compare preferences of both patients as well as referring physicians regarding publicly available hospital quality information

**Methods:**

Based on systematic literature reviews as well as qualitative analysis, two discrete choice experiments (DCEs) were applied to elicit patients’ and referring physicians’ preferences. The DCEs were conducted using a fractional factorial design. Statistical data analysis was performed using multinomial logit models

**Results:**

Apart from five identical attributes, one specific attribute was identified for each study group, respectively. Overall, 322 patients (mean age 68.99) and 187 referring physicians (mean age 53.60) were included. Our models displayed significant coefficients for all attributes (*p* < 0.001 each). Among patients, “Postoperative complication rate” (20.6%; level range of 1.164) was rated highest, followed by “Mobility at hospital discharge” (19.9%; level range of 1.127), and ‘‘The number of cases treated” (18.5%; level range of 1.045). In contrast, referring physicians valued most the ‘‘One-year revision surgery rate’’ (30.4%; level range of 1.989), followed by “The number of cases treated” (21.0%; level range of 1.372), and “Postoperative complication rate” (17.2%; level range of 1.123)

**Conclusion:**

We determined considerable differences between both study groups when calculating the relative value of publicly available hospital quality information. This may have an impact when calculating aggregated composite measures based on consumer-based weighting.

**Supplementary Information:**

The online version contains supplementary material available at 10.1007/s10198-023-01650-2.

## Introduction

Public reporting of performance information encompasses data, publicly available or available to a broad audience free of charge or at a nominal cost, about a health-care structure, process, or outcome at any provider level (e.g., hospitals]) [1]. The overall aim of public reporting is to improve health-care quality by both stimulating quality improvement on the provider level (“Improvement Through Changes in Care”) and also by helping patients, referring physicians, and other consumers select the “right” provider (“Improvement Through Selection”) [2, 3]. Therefore, hospital report cards (HRCs) publicly display quality-related information about hospitals and enable hospital comparisons [4]. However, studies have shown heterogeneous results regarding the impact of HRCs on the choice behavior of consumers [5–10]. To increase the uptake of HRCs of consumers, HRCs should present the information that consumers value most when making hospital choices [11]. Besides, it should be attempted to limit the richness of consumers’ choice sets, i.e., the number of hospitals as well as the variety of quality measures [12].

In this context, one promising strategy is to provide customized information that both reflects the preferences of consumers and reduces the amount of data by aggregating individual performance measures into summary scores (i.e., composite measures) [11, 13–17]. For example, a recent study has shown that the introduction of summary measures on Nursing Home Compare—a web-based guide detailing quality of care at over 17,000 Medicare- or Medicaid-certified nursing homes in the United States—was associated with a significant change in consumer demand for low- and high-scoring facilities [18]. So far, composite measures have been published in several countries such as the United States, the UK, and Germany (e.g., Hospital Compare, Nursing Home Compare, NHS Choices, US News Best Hospital, AOK Hospital Navigator) [17–19].

There are different approaches to calculate aggregated composite measures such as opportunity-based weights, numerator-based weights, all-or-none measures, expert panels, and consumer-based weighting [16]. As shown, consumer-based weighting aligns mostly closely with patient-centered care and should, therefore, be prioritized [16]; for this purpose, it is crucial to learn more about consumers’ preferences when choosing a hospital. So far, current composite measure approaches do not consider consumer preferences in more detail when calculating summary scores. For example, the overall star rating system for hospitals for the Centers for Medicare and Medicaid Services (CMS) in the United States—which is publicly displayed on Hospital Compare—is calculated by taking the weighted average of the hospital’s scores across five areas of quality (i.e., mortality, safety of care, readmission, patient experience, and timely and effective care). Therefore, the relative weight for mortality, safety, readmission, and patient experience is worth 22% each, and timely and effective care is worth 12% of the overall score [20].

In addition, most research has been conducted to investigate whether patients—as the main target group of HRCs—use publicly reported quality information to search for and select hospitals [21–23]. However, less information is available regarding whether publicly available quality information plays a role from the referring physicians’ perspective [24, 25]. This seems to be surprising since most patients trust their referring physicians’ recommendation regarding what hospital to choose [25, 26]. Therefore, referring physicians should be regarded as another major target group of HRCs so as to direct patients to well-performing hospitals and to increase the impact of public reporting [24, 27].

In this context, the present study aims to identify the most important publicly reported hospital quality information for hospital choice for elective hip replacement surgery and to determine their relative importance among both patients as well as referring physicians. We focused on elective hip replacement surgery for the following reasons: first, we aimed to address one procedure that is both standard for public reporting initiatives as well as included in German hospital quality assurance initiatives [28, 29]. Second, non-acute procedures should be prioritized since public reporting aims to support patients, referring physicians, and other consumers to select the “right” provider. [2, 3] Third, elective hip replacement surgery is one of the most frequently conducted procedures in Germany; for example, 160,910 surgeries were performed in 1,254 hospitals in 2020 [30]. The results might help us learn how to weight different quality measures when calculating composite measures. In more detail, we sought to address the following three questions: (1) what is the essential publicly available hospital quality information for choosing a hospital for elective hip replacement surgery from the perspective of patients and referring physicians? (2) How do patients and referring physicians’ rate different publicly available hospital quality information? (3) What relative importance do both patients and referring physicians assign to different quality information?

## Materials and methods

This study used a mixed methods approach. After performing a systematic literature review, we conducted qualitative research methods (i.e., semi-structured interviews) to identify and select the most important quality measures for choosing a hospital for hip replacement surgery from the perspective of both patients and referring physicians. Based on this, two separate discrete choice experiments (DCEs) were developed and performed to elicit and compare patients’ and referring physicians’ preferences for all relevant hospital choice characteristics and to determine the relative value of each quality measure. DCEs are increasingly used in the health-care context to inform on consumer preferences for health-care services such as hospital choice [31]. DCEs are a stated preference method that use (survey) data to systematically elicit individuals’ preferences based on a series of hypothetical choice scenarios (termed choice sets) [32]. Two key economic theories (i.e., Random Utility Theory and Lancaster’s Theory [33, 34]) suggest that respondents choose the option from each choice set which provides them with the most satisfaction or ‘‘utility” [32]. Based on the decisions, DCEs can help understand which characteristics (termed attributes) are preferred by consumers and determine the relative value of each attribute. [32, 35]

### Study design

The design and analysis of both DCEs were based on standardized research practices for undertaking conjoint analysis of the ISPOR Conjoint Analysis Good Research Practices Task Force [36–38]. Ethics approval for both studies was obtained from the Friedrich-Alexander-University Erlangen-Nuremberg Ethics Board (196_19 B). Informed consent was obtained from each study participant.

### Systematic search procedure

First, we conducted two systematic search procedures on Medline (via PubMed) and the Cochrane Library to identify studies which aimed to identify relevant criteria for hospital choice for hip replacement surgery from the perspective of both patients and referring physicians. The searches were carried out in September 2020 and aimed to identify English and German language literature published since 2010. In addition, reference lists of identified research articles were screened for further articles. The reviews were complied with the Guideline from the Cochrane Collaboration. [39]

The search strategy addressing the *perspective of patients* was segmented into three components. The first component referred to hospitals (e.g., hospital, clinic), the second component referred to choice (e.g., choice, selection), and the third component addressed hip replacement surgery (e.g., hip, coxarthrosis). As a result, 5,510 potentially relevant papers were identified. After eliminating duplicates and judging titles and abstracts in a first step as well as full papers in a second step, ten studies were considered relevant [40–49]. In sum, 73 individual criteria were derived from the 10 studies. We added four further quality measures from the German hospital quality report administered by the Institute for Quality Assurance and Transparency in Healthcare (IQTIG). This led to a final sample of 77 quality measures which were then merged into 23 more general measures (e.g., the following three individual criteria “28-day mortality rate (%)”, “28-day mortality rate following discharge”, and “Postoperative mortality” were merged into the category “Mortality rate”). Afterward, we excluded ten criteria which are not available in the German hospital sector for public reporting purposes (e.g., waiting time, 28-day emergency readmission rate), so that 13 criteria remained for the next step (See Supplemental Material 1).

The search strategy addressing the *perspective of referring physicians* was based on previously published literature and slightly modified [50]. As a result, 2,246 potentially relevant papers were identified. After eliminating duplicates and judging titles and abstracts in a first step as well as full papers in a second step, 16 studies were considered relevant [25, 51–65]. In sum, 39 criteria were derived from those studies. Then we excluded 20 criteria that are not publicly available (see above) (e.g., waiting times, MRSA events), so that 19 criteria remained.

### Qualitative steps (semi-structured interviews)

In total, we qualitatively surveyed (January 2021 to February 2021) 20 randomly selected hip replacement surgery patients (mean age 63.9 years, 55% female) from a German statutory health insurance who had undergone elective hip arthroplasty surgery within 3 years prior to answering the survey as well as 15 referring physicians (mean age 56.3 years, 20% female). Written informed consent was obtained from all participants. Respondents were sent a short survey through postal mail before conducting the semi-structured interviews to learn more about (the) past hospital choice(s) and the importance of the 13 resp. 19 criteria for choosing a hospital. After receiving the short survey, semi-structured interviews were conducted to explore the stated preferences, to determine the most relevant criteria as well as corresponding levels, to clarify the wording, and to evaluate the comprehensibility of hypothetical choice tasks for the DCE (see below). In particular, we evaluated the comprehensibility of the labels used for the attributes and their corresponding descriptions to assure the intended interpretation of the explanations. Based on all steps, five attributes were derived which were of major importance for choosing a hospital from the perspective of both patients and referring physicians which were as follows: the rate of confirmed diagnosis prior the surgery, the certification as an Endoprosthetics Center, the number of cases treated, the rate of postoperative complications, as well as the rate of mobility at hospital discharge. This was supplemented with the assessment of actions to prevent falls of patients for patients and the 1-year revision surgery rate for referring physicians, respectively (Table 1). Individuals who completed the qualitative study received 50 Euro.

**Table 1:** Summary of attributes and levels used in the discrete choice experiments (DCE)

Attribute	*Description* and levels given in DCE questionnaire
Confirmed diagnosis rate^$,#^	*Hip replacement primary implantation with fulfilled indication criteria*
	Quality targets reached
	Quality targets not reached
	No assessment intended resp. results not (yet) available
EndoCert certificate^$,#^	*Hospitals which meet requirements of the EndoCert certification system*
	No certificate
	Certified EndoProstheticsCenter (EPZ)
	Certified EndoProstheticsCenter of Maximum Care (EPZmax)
The number of cases treated^$,#^	*The number of total elective hip replacement surgeries in a hospital*
	84 patients (below average)
	148 patients (average)
	230 patients (above average)
Postoperative complication rate^$,#^	*Postoperative specific complications after hip replacement surgery (e.g., infections, bleeding, thromboembolism)*
	Quality targets reached
	Quality targets not reached
	No assessment intended resp. results not (yet) available
Mobility at hospital discharge^$,#^	*Functional mobility status on discharge after hip replacement surgery*
	Quality targets reached
	Quality targets not reached
	No assessment intended resp. results not (yet) available
Prevention of falls measures^$^	*Hospitals should be active in minimizing fall risk among patients who have undergone hip replacement surgeries*
	Quality targets reached
	Quality targets not reached
	No assessment intended resp. results not (yet) available
1-year revision surgery rate^#^	*Percentage of patients who underwent revision surgery within one year after initial surgery*
	1.41% (better than average)
	2.48% (average)
	3.89% (lower than average)

^$^This attribute applies for patients

^#^This attribute applies for referring physicians

### Quantitative steps

#### The survey instrument

Both survey instruments were similar and consisted of four parts. First, we asked for general sociodemographic information (e.g., age, gender) before collecting information about the experience with HRCs when selecting hospitals in the second part. Second, respondents were presented with publicly available hospital quality information items (see above) as well as short descriptions and were asked to rate each item on a 1–5 scale (1 = not all important; 5 = very important). Furthermore, we asked the respondents to select the single most important information item for the hospital choice. Afterward, all respondents were asked to respond to the DCE survey block. Both DCE experiments were designed using Sawtooth Software Lighthouse Studio 9.11.0.

**Fig. 1:**
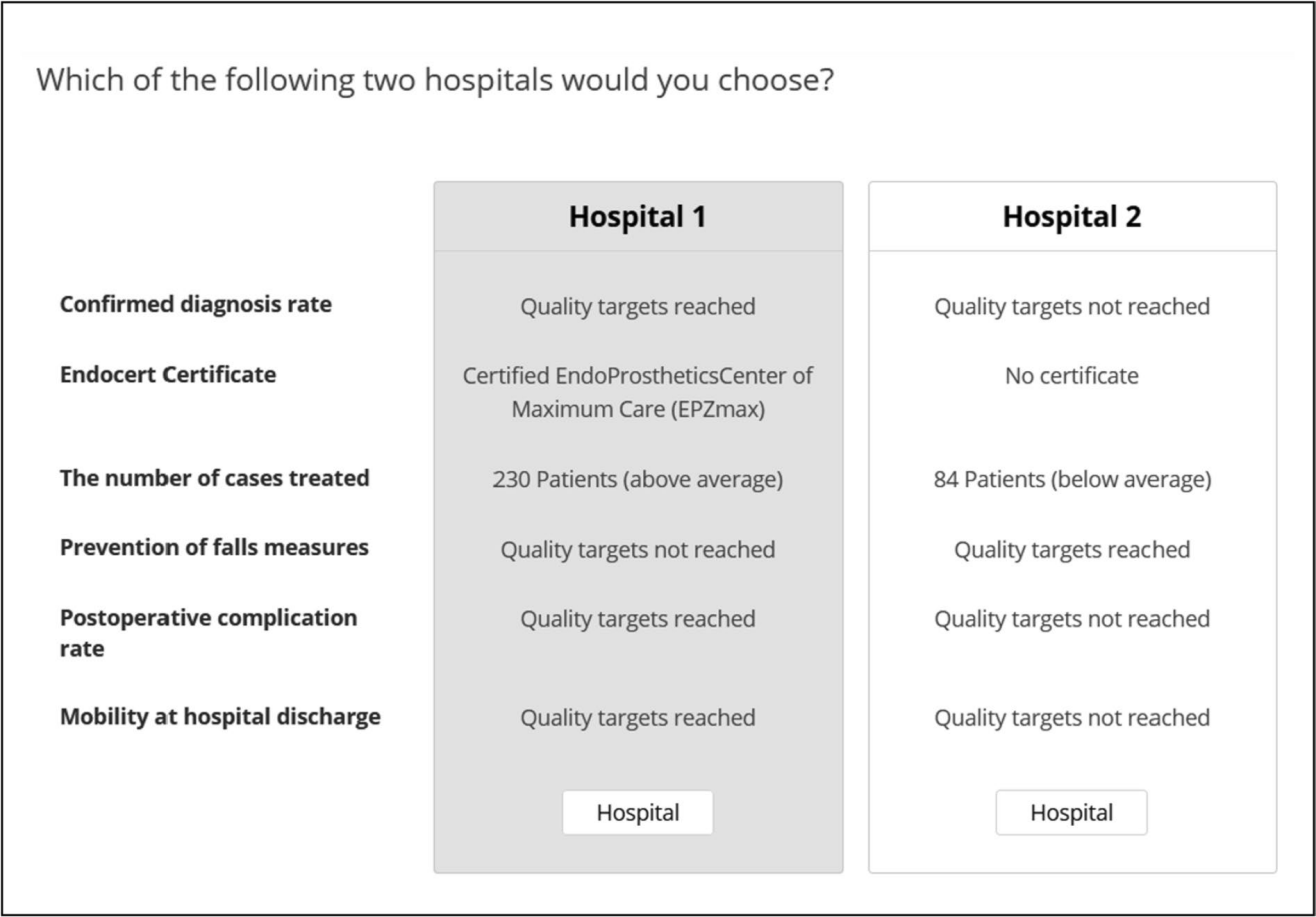
Example choice task for patients

Instead of ranking or rating different quality measures, as is done in traditional importance elicitation formats, DCEs perform a pairwise comparison of hypothetical alternatives (i.e., differently configured hospitals) and ask the participants to choose between them [66]. Both experiments were designed as a full profile design (i.e., each choice set included all six attributes) and were generated using the balanced overlap method so as to achieve standard errors below 0.05 for main effect utilities and 0.10 or smaller for interaction effects and the highest D-efficiency score [67, 68]. We created 10 versions (termed blocks) of the DCE, each containing 10 choice tasks, thus generating 100 unique choice tasks (fractional factorial design). Choice tasks were generated that maximized both balance (i.e., meaning each level appears with the same frequency) and orthogonality (i.e., meaning each pair of levels appears with the same frequency across each pair of attributes) [69]. The choice sets in both surveys were designed as forced-choice tasks; this means that respondents had to choose one of two hypothetical hospitals by making trade-offs between attributes and their levels. This method offers practical advantages such as closeness to reality as trade-off decisions are part of everyday life [66]. As stated above, hospitals differed in six attributes with three levels each. In DCE research, four to eight attributes per choice set are seen as appropriate [70, 71]. Figure 1 provides one hypothetical choice task as an example. The questionnaire was pilot tested for clarity and understanding with 15 hip replacement patients as well as 15 referring physicians and slightly modified accordingly.

#### The survey sample

Final questionnaires were sent through postal mail to 1000 randomly selected people from a German statutory health insurance (AOK Lower Saxony) who had undergone elective hip arthroplasty surgery within 3 years prior to answering the survey. One reminder was sent out 2 weeks after the initial invitation. Furthermore, we purchased a database containing contact information (e.g., postal address, email) for orthopedists in the German outpatient sector from a commercial provider (ArztData AG). This database covers about 88% of all orthopedists in the German outpatient sector [72]. We used a sequential mixed-mode strategy to achieve high response rates. In a first step, 1,650 referring orthopedists were contacted via email, which contained a link to participate online (web-based survey). After 1 week, a first reminder was sent out. In a second step, the remaining orthopedists were contacted via postal mail and received a printed version of the survey; after 2 weeks, we sent out a second reminder. The surveys were conducted between March and June 2021 in the German language. As an incentive, respondents received a payment of approximately €50.

#### Sample size

Johnson’s often-used rule-of-thumb calculates a sample of 75 participants for a DCE having our design specifications (i.e., ten choice tasks per respondent, two alternatives, three levels per attribute as maximum) [73]. This sample size was discussed as being the lowest limit for main effects estimation. However, we aimed at doubling this number (i.e., to include at least 150 participants) following more advanced recommendations for statistical robustness [74, 75].

### Statistical methods

Analyses of general survey questions were performed using SPSS (IBM Corp. Released 2019. IBM SPSS Statistics for Windows, Version 26.0. Armonk, NY: IBM Corp). Descriptive statistics (i.e., means for continuous variables, percentages for categorical variables) were used to examine the demographic and experience variables.

For analyzing the DCEs, we performed multinomial logit models using R (Version 4.2.2) and the corresponding mlogit-package by Croissant (2020) [76]. We constructed the data sets in long format. Thus, the data set about patients included 6,440 observations (322 patients × 10 choice tasks per subject × 2 alternatives per choice set). Similarly, the data set for physicians comprised 3,740 observations (187 physicians × 10 choice tasks per subject × 2 alternatives per choice set). As for data encoding, we started from assigning each variable equidistant numerical values of scale {0; 1; 2} as described by Mühlbacher et al. [77]. For example, regarding the attribute “Confirmed diagnosis rate”, we designated the value {0} to “Quality targets not reached”, value {1} to “No assessment intended resp. results not (yet) available” (please note: due to the implicit uncertainty of this alternative, we assumed that the absence of results lay between the certain answers “targets reached” and “targets not reached”), and value {2} to “Quality targets reached”. Afterward, we repeated the estimations treating the attributes as categorical variables. Both approaches provided qualitatively very similar results, whereby the estimates from categorical attributes could be shown to be more precise.

We employed effect coding by following standard guidelines [38, 78]. Therefore, we expressed every attribute as *n*-1 variables where n is the number of levels. Each variable corresponds to one level, while the omitted level serves as the reference. If the variable matches the level presented in the corresponding profile, we assigned value {1}. If the level in the corresponding profile equals another non-omitted level, we assigned value {0}. If the level in the corresponding profile is the omitted level, we assign value {− 1}. For example, the attribute “Confirmed diagnosis rate” was operationalized by three possible levels: “Quality targets reached”, “Quality targets not reached”, “No assessment intended resp. results not (yet) available”. As reference level, we chose “No assessment intended resp. results not (yet) available”. Hence, we constructed two variables; the first covered the level “Quality targets reached”, the other one the level “Quality targets not reached”. If the given occurrence equals the reference, we filled both variables with {-1}, otherwise we assigned values {1} or {0}. As reference categories, we assigned “Certified Endoprosthetics Center of Maximum Care (EPZmax)” for the attribute “Endocert Certificate”, middle positions for the attributes “Number of cases treated” and “One-year revision surgery rate”, and finally “No assessment intended resp. results not (yet) available” for all remaining attributes. We chose effect coding over dummy coding since with dummy coding, the parameter estimate for the (omitted) baseline category is equal to zero and cannot be recovered. Thus, the estimates of the other levels are only relative to the benchmark level. Instead, with effect coding, we can extract the parameter of the reference category from the negative sum of the included categories and the standard error from the covariances of the included categories. This enables us to compare all levels of the attributes against the corresponding mean value with the sign of the coefficients indicating a positive or negative impact compared to the mean of the attribute and the magnitude specifying the size of the effect [38, 78, 79]. In all approaches, we applied Akaike Information Criterion (AIC), Bayesian Information Criterion (BIC), and log-likelihood value (LL) to identify the best fitting model. The relative importance of each attribute was determined based on each attribute’s coefficient range. This means that we calculated the difference between the coefficients of the highest and lowest level of each attribute and expressed this as a share of the total range across all attributes.

## Results

### Sample characteristics

Overall, 378 patients (37.8% response rate) and 267 referring orthopedists (16.2% response rate) participated in our study and returned the survey. The following analysis reports on the 322 patients and 187 orthopedists who fully completed the DCE component of the questionnaire and provided consistent responses (see Table 2 for the demographic characteristics of both study groups). The mean age of patients was 68.99 (SD 10.44) years. Slightly more than half of patients (51.6%) were female, and most patients (53.7%) stated secondary general school or less as the highest educational level. Approximately one in four patients was aware of HRCs when answering the survey (26.1%). The mean age of participating orthopedists was 53.60 (SD 8.14) years, a large majority of respondents were male (90.4%), and 39 respondents (21.2%) indicated prior HRC experience in searching for a hospital. Almost three out of four surveyed referring orthopedists (72.7%) stated that they perceived substantial differences in the quality of care between hospitals.

**Table 2:** Key characteristics for both patients (*n* = 322) and referring physicians (*n* = 187)

	Patients (*n* = 322)		Referring physicians (*n* = 187)	
	Mean (SD) or *n*	Range or %	Mean (SD) or *n*	Range or %
Age				
Mean (SD)	68.99 (10.44)	35–89	53.60 (8.14)	33–73
Gender				
Male	151	46.9%	169	90.4%
Female	166	51.6%	18	9.6%
Missing	5	1.6%	0	0.0%
Educational attainment				
Secondary general school or less	173	53.7%	n.a	n.a
Intermediate secondary school	93	28.9%	n.a	n.a
(Technical) University entrance qualification	38	11.8%	n.a	n.a
Missing	18	5.6%	n.a	n.a
Chronic conditions				
Any chronic condition	141	43.8%	n.a	n.a
No chronic condition	159	49.4%	n.a	n.a
Missing	22	6.8%	n.a	n.a
Health Status				
Good or better	168	52.2%	124	66.3%
Satisfactory	122	37.9%	51	27.3%
Bad or worse	26	8.1%	12	6.4%
Missing	6	1.9%	0	0.0%
Perceived differences in the hospital quality				
Big differences	81	25.2%	136	72.7%
Small differences	89	27.6%	48	25.7%
No differences	23	7.1%	2	1.1%
I don`t know	122	37.9%	1	0.5%
Missing	7	2.2%	0	0.0%
Knowledge of hospital report cards				
Yes	84	26.1%	39	21.2%
No	188	56.4%	145	78.8%
Missing	50	15.5%	0	0.0%
Practice type				
Single physician practice	n.a	n.a	65	34.8%
Group physician practice	n.a	n.a	76	40.6%
Outpatient-based health-care center	n.a	n.a	40	21.4%
Other	n.a	n.a	6	3.2%
Attending doctor (i.e., with hospital affiliation)				
Yes and I perform the surgery myself	n.a	n.a	40	21.4%
Yes but I do not perform the surgery myself	n.a	n.a	21	11.2%
No	n.a	n.a	124	66.3%
Missing	n.a	n.a	2	1.1%

### Descriptive rating and ranking results

The results regarding the importance of different information items for the hospital choice—on a 1–5 scale (1 = not all important; 5 = extremely important)—showed that patients rated confirmed diagnosis (hip surgery) rate (4.65 ± 0.80), mobility at hospital discharge (4.60 ± 0.73), and complication rate (4.54 ± 0.81) as most important (see Supplemental material 2). In contrast, referring physicians rated complication rate (4.19 ± 1.06), the number of cases treated (4.09 ± 0.98), and the 1-year revision surgery rate (4.00 ± 1.21) as most important. Furthermore, the survey results for the single most important information item for the hospital choice revealed confirmed diagnosis (hip surgery) rate (44.5%) as well as the number of cases treated (35.9%) as most relevant from the perspective of patients and referring physicians, respectively.

### Results of the discrete choice experiments

The applied models displayed significant coefficients for all attributes included in both analyses (*p* < 0.001 each). This means that, independent of the placement, all attributes were relevant to the decision of both patients and referring physicians (Supplemental material 3). [Please also note that some mean levels could not been shown to be statistically significant; for example, the level “No assessment intended/results not (yet) available” did not reach statistical significance in case of the “Confirmed diagnosis rate” and the “Mobility of hospital discharge”.] Among patients, the highest preference weights for hospital choice were calculated for “Postoperative complication rate” (coef.: 0.560; SE: 0.037), ‘‘Mobility at hospital discharge” (coef.: 0.551; SE: 0.036), and ‘‘The number of cases treated” (coef.: 0.547; SE: 0.036). The fourth position was occupied by ‘‘Confirmed diagnosis (hip surgery) rate’’ (Coef.: 0.437; SE: 0.033), followed by ‘‘Prevention of falls measures’’ (Coef. 0.431; SE: 0.035), and ‘‘Endocert Certificate’’ (Coef. 0.341; SE: 0.033). In contrast, referring physicians showed a clear preference for a decrease in ‘‘One-year revision surgery rate’’ (coef.: 1.014; SE: 0.053). The following positions were occupied by “The number of cases treated” (coef.: 0.696; SE: 0.051) and “Postoperative complication rate” (coef.: 0.546; SE: 0.050). Here, the “Confirmed diagnosis (hip surgery) rate” (coef.: 0.390; SE: 0.052), “Endocert Certificate’’ (coef.: 0.310; SE: 0.047), and the ‘‘Mobility at hospital discharge’’ (coef.: 0.236; SE: 0.046) were less important for referring physicians. The same order can be derived from the calculation of the relative importance of all attributes for both study groups. The relative importance was calculated based on each attribute’s coefficient range (i.e., the difference between the coefficients of the highest and lowest level of each attribute) expressed as a share of the total range across attributes (Table 3). Table 3 also presents the odds ratio (OR) for each level to analyze its impact on the hospital choice. For example, the likelihood of voting a hospital with a successful result regarding the “Confirmed diagnosis rate” (i.e., quality targets reached) is 1.535 times higher than the probability of choosing a hospital with the mean of “confirmed diagnosis rate”.

**Table 3:** Estimated parameters of multinomial logit (MNL) models

Attributes and levels	Patients (*n* = 322)							Referring physicians (*n* = 187)						
	Coeff	OR	SE coeff	95% CI		p	Difference (highest—lowest level)	Coeff	OR	SE coeff	95% CI		p	Difference (highest—lowest level)
Confirmed diagnosis rate^$,#^														
Quality targets reached	0.429	1.535	0.040	0.350	0.507	< .001	0.856	0.454	1.575	0.058	0.340	0.568	< .001	0.800
No assessment intended/results not (yet) available	− 0.001	0.999	0.039	− 0.078	0.076	0.977		− 0.108	0.897	0.056	− 0.218	0.001	0.051	
Quality targets not reached	− 0.427	0.652	0.038	− 0.503	− 0.352	< .001		− 0.346	0.708	0.062	− 0.467	− 0.224	< .001	
^#^Endocert Certificate^$,^														
No certificate	− 0.390	0.677	0.040	− 0.469	− 0.311	< .001	0.646	− 0.446	0.640	0.058	− 0.561	− 0.331	< .001	0.718
Certified EndoProstheticsCenter	0.135	1.144	0.040	0.055	0.214	0.001		0.174	1.191	0.054	0.068	0.281	0.001	
Certified EndoProstheticsCenter of Maximum Care	0.256	1.291	0.039	0.179	0.332	< .001		0.272	1.312	0.059	0.155	0.388	< .001	
The number of cases treated^$,#^														
84 patients (below average)	− 0.616	0.540	0.040	− 0.695	− 0.537	< .001	1.045	− 0.676	0.509	0.058	− 0.791	− 0.560	< .001	1.372
148 patients (average)	0.188	1.207	0.039	0.112	0.264	< .001		− 0.020	0.980	0.056	− 0.131	0.090	0.716	
230 patients (above average)	0.428	1.535	0.044	0.341	0.516	< .001		0.696	2.006	0.058	0.581	0.811	< .001	
Postoperative complication rate^$,#^														
Quality targets reached	0.622	1.863	0.042	0.540	0.704	< .001	1.164	0.600	1.823	0.061	0.481	0.720	< .001	1.123
No assessment intended/results not (yet) available	− 0.080	0.923	0.039	− 0.157	− 0.003	0.040		− 0.078	0.925	0.057	− 0.190	0.034	0.170	
Quality targets not reached	− 0.542	0.582	0.044	− 0.628	− 0.455	< .001		− 0.522	0.593	0.057	− 0.636	− 0.409	< .001	
Mobility at hospital discharge^$,#^														
Quality targets reached	0.597	1.817	0.043	0.513	0.682	< .001	1.127	0.344	1.410	0.057	0.231	0.456	< .001	0.536
No assessment intended/results not (yet) available	− 0.068	0.935	0.039	− 0.145	0.010	0.085		− 0.192	0.826	0.061	− 0.313	− 0.071	0.002	
Quality targets not reached	− 0.530	0.589	0.041	− 0.610	− 0.449	< .001		− 0.152	0.859	0.056	− 0.263	− 0.042	0.007	
Prevention of falls measures^$^														
Quality targets reached	0.458	1.581	0.039	0.381	0.535	< .001	0.822	n.a	n.a	n.a	n.a	n.a	n.a	n.a
No assessment intended/results not (yet) available	− 0.094	0.911	0.040	− 0.172	− 0.016	0.018		n.a	n.a	n.a	n.a	n.a	n.a	
Quality targets not reached	− 0.364	0.695	0.042	− 0.447	− 0.281	< .001		n.a	n.a	n.a	n.a	n.a	n.a	
1-year revision surgery rate^#^														
1.41% (better than average)	n.a	n.a	n.a	n.a	n.a	n.a	n.a	0.977	2.657	0.094	0.791	1.163	< .001	1.989
2.48% (average)	n.a	n.a	n.a	n.a	n.a	n.a		0.035	1.035	0.159	− 0.279	0.348	0.827	
3.89% (lower than average)	n.a	n.a	n.a	n.a	n.a	n.a		− 1.012	0.363	0.097	-1.203	− 0.821	< .001	
Model constant (Intercept)	− 0.309	0.734	0.047	− 0.402	− 0.216	< .001		n.a	n.a	n.a	n.a	n.a	n.a	

*OR* odds ratio (OR = exp(coeff)); *SE* standard errors, *CI* confidence interval

^$^This attribute applies for patients

^#^This attribute applies for referring physicians

**Fig. 2:**
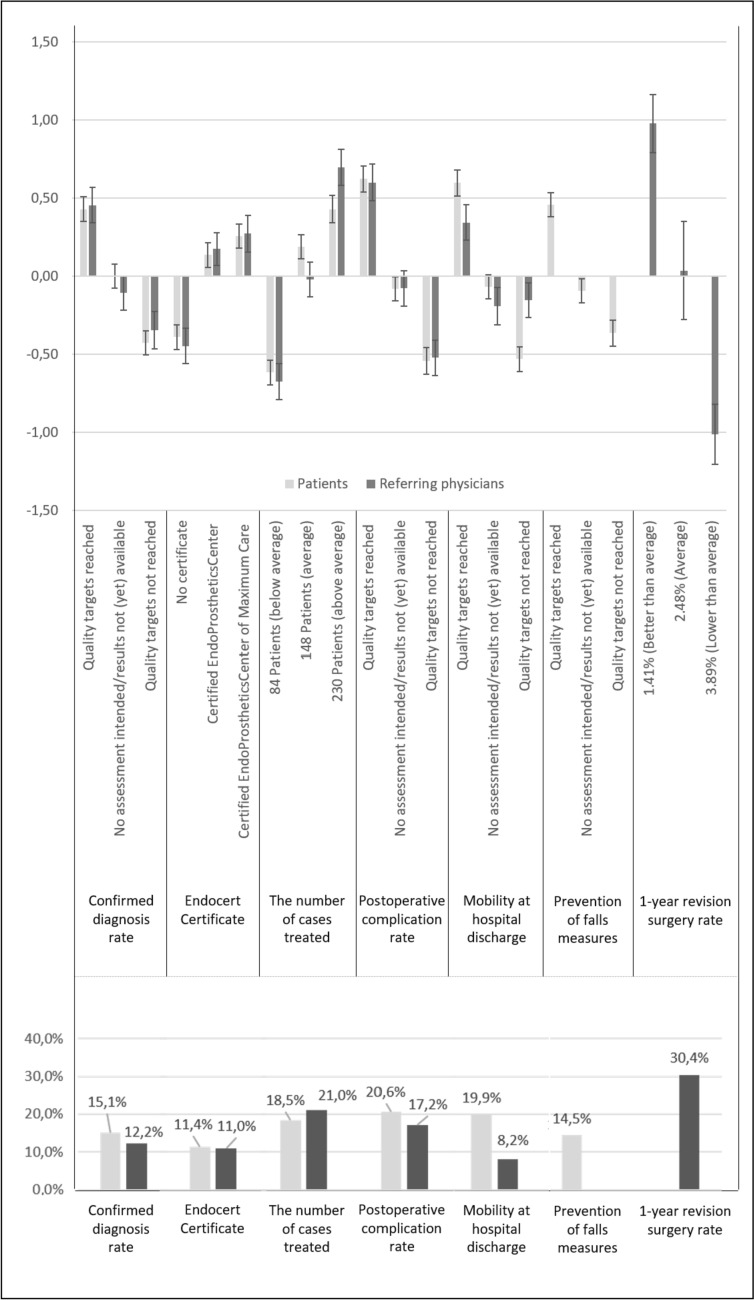
Graphic display of level estimates with 95% CI (discrete choice experiment) and mean relative importance of attributes for both subgroups for the hospital choice

As shown in Fig. 2, the preference pattern of patients showed a relatively small range of results. The following three attributes, “Postoperative complication rate” (20.6%; level range of 1.164), “Mobility at hospital discharge” (19.9%; level range of 1.127), and “The number of cases treated” (18.5%; level range of 1.045) were weighted highest. The lowest weight was detected for “Endocert Certificate” (11.4%; level range of 0.646). In contrast, the preference pattern of referring orthopedists showed larger differences in the relative importance of presented quality information measures. Here, the “One-year revision surgery rate” was identified as the main dominant attribute (30.4%; level range of 1.989). Furthermore, “The number of cases treated” (21.0%; level range of 1.372) and “Postoperative complication rate” (17.2%; level range of 1.123) were relatively highly weighted. Here, the lowest weight was detected for “Mobility at hospital discharge” (8.2%; level range of 0.536).

## Discussion

The objective of the present study was to identify the most important publicly reported hospital quality information for hospital choice and to determine their relative importance among both former patients and referring physicians in Germany. The results might assist us in learning how to weight different quality measures when calculating aggregated patient-centered composite measures.

Based on our results, several conclusions can be drawn. First, patients and referring physicians selected similar publicly available quality information for the hospital choice. However, it should be mentioned that we focused on publicly available quality information and did not include information which is not available for public reporting purposes or which is not routinely collected in the health-care system in our experiments. Therefore, we cannot exclude that the integration of such information might have led to different findings. For example, Geraedts and colleagues showed that many surgical patients decided in favor of a hospital because of the trust they had built up through previous treatment in the hospital, which they experienced as satisfactory [80].

Second, HRCs should present the information that consumers value most when making hospital choices. At least five of the seven most relevant publicly reported quality information items are displayed on most German HRCs (e.g., Weisse Liste, AOK Hospital Navigator) [28]. This is mainly due to the fact that those information items are part of the German external hospital quality assurance system whose results are made available for public reporting purposes [81]. In contrast, the certification as a Certified Endoprosthetics Center is only presented on a few HRCs so far (e.g., Weisse Liste). Besides, the 1-year revision rate is only presented on the hospital navigator of the Allgemeine Ortskrankenkasse (AOK) [81]. The AOK is a major provider of statutory health insurance in the German health-care system and calculates further quality information based on claims data [29]. Following this, providers of HRCs might compare whether the most relevant quality information is already included on its own HRC. If not, providers might consider adjusting the content as a first step toward more consumer orientation.

Third, the study’s DCE-based findings appear broadly consistent with the results from the rating-based survey. For example, the preference pattern of patients showed a relatively small range of results. While the rating-based findings (on a scale of 1–5) varied between 4.09 ± 1.03 (Endocert Certificate) and 4.65 ± 0.80 (confirmed diagnosis hip surgery rate), the DCE-based relative importance values ranged between 11.4% (“Endocert Certificate”) and 20.6% (Postoperative complication rate), respectively. In both cases, “Endocert Certificate” was the least important attribute for the hospital choice while “Complication rate” and “Mobility at hospital discharge” were among the most important attributes for the hospital choice. However, the ranking-based results showed slightly different findings. While the confirmed diagnosis (hip surgery) rate (44.5%) was shown to be the single most important information item for the hospital choice, the DCE-based findings revealed a relative importance value of 15.1%. In addition, the overall results regarding the preference pattern of referring physicians were shown to be very consistent, regardless of the methodology. For example, the three attributes “The number of cases treated”, “Complication rate”, and “1-year revision surgery rate” showed the strongest results. In contrast, the “Confirmed diagnosis (hip surgery) rate”, “Endocert Certificate”, and “Mobility at hospital discharge” were shown to be less important.

Fourth, the results of this study could be used for the calculation of composite measures [11, 13–17]. Therefore, we applied a consumer-based weighting approach as recommended and determined the relative weight of relevant information [16]. For example, the preference pattern of referring orthopedists showed the “One-year revision surgery rate” to be most important with a relative weight of 30.4%. The challenge now is to convert the hospital quality results into (numeric) scores for calculating the composite measure. As stated by van Til and colleagues [82], the overall preference for alternatives (i.e., hospitals) might be estimated based on the sum of the part-worth utilities for the selected level of all attributes. For example, a clinic certified as an Endoprosthetics Center of Maximum Care (coef.: 0.272), with a below-average 1-year revision surgery rate (coef.: 0.977), an above-average number of cases (coef.: 0.696), that also achieves the statutory quality targets regarding confirmed diagnosis rate (coef.: 0.454), postoperative complication rates (coef.: 0.600), and mobility at hospital discharge (coef.: 0.344), would have an overall score of 3.343 from the perspective of referring physicians. In contrast, an overall score of − 3.194 would be calculated for a hospital that achieves the most negative level expressions for each attribute. [Please consider this example to be simplified. A more advanced approach would consider the corresponding utility function to estimate the expected overall utility of each alternative (i.e., hospital).] Based on this, hospitals could be ranked and then grouped into several performance groups. Therefore, our study has shown that consumer preferences might differ substantially between target groups and each preference structure should be considered individually when developing rating systems based on composite measures. However, it should also be noted that our approach has certain limitations that must be considered carefully. For example, the attribute levels are used to operationalize the alternatives included in the choice sets. Therefore, it is important to select attributes and corresponding levels that properly describe the health-care product or service (i.e., the hospitals). If the levels are not defined in the appropriate range, the estimated coefficients could be biased [83]. Other methodological issues might also have an impact on the results derived by DCE studies and should be considered carefully [83, 84].

Finally, the results might serve as the basis for improved physician–patient communication. The findings might increase the mutual understanding of hospital choice preferences. In particular, referring physicians might better understand what patients actually want and which hospital they would prefer. They might have an important "agent" function in the hospital choice especially for patients with little time prior to admission and those who do not decide themselves [85]. Therefore, by understanding factors that influence patients’ hospital choice decisions, referring physicians may become more sensitive to patients’ preferences, which may have a positive effect on the process and outcomes of shared decision making [86]. This would increase the likelihood that patients may be satisfied with both the hospital choice process and the hospital choice itself [87]. Our study makes some contributions to such understanding, since it shows that, in contrast to referring physicians, the 1-year revision rate did not influence patients’ stated preferences, whereas the prevention of falls measures did.

As with any study, there are several limitations that have to be considered. First, it is important to mention that this study was conducted in Germany and might be of limited relevance for other countries. Nevertheless, the results presented in this paper are of interest for all countries with public reporting initiatives, such as the United States and others. For example, Hospital Compare in the United States already provides an overall composite measure for hospitals but does not consider preferences of patients and referring physicians in detail [20]. Second, it must be noted that the selected attributes for the DCEs referred to publicly available quality measures. Therefore, we cannot exclude that other quality measures are more relevant from the perspective of both study groups. Nevertheless, it seems reasonable for our study purpose to follow the approach used. In comparable studies, very similar approaches have been applied [47]. Third, our study analyses stated that preferences of the respondents to hypothetical scenarios and their actual responses may differ [47]. Fourth, the sample of surveyed referring physicians (*n* = 187) was smaller than that of patients surveyed (*n* = 322). In this regard, it should be noted that we aimed to reach a minimum number of 150 participants in both studies to meet Johnson’s often-used rule-of-thumb mentioned above [73], taking into account more advanced recommendations for statistical robustness [74, 75]. Following this, we were able to achieve our set target regarding the recruitment in both studies. Finally, the comprehensibility of the study attributes and corresponding levels is crucial for conducting DCEs and analyzing the corresponding data. During our study, we aimed to address this topic by means of different approaches. First, we qualitatively interviewed 20 patients who had undergone elective hip arthroplasty surgery within 3 years prior to answering the survey. Thereby, we evaluated the comprehensibility of the labels used for the attributes and their corresponding descriptions in particular to assure the intended interpretation of the explanations. Based on those interviews, slight modifications were made to eliminate remaining uncertainties. Second, the respondents had to work with the presented study attributes and corresponding levels before conducting the choice-based part of the DCE. Both approaches might have positively influenced the comprehensibility of the study attributes and levels. In addition, the surveyed patient sample consisted of patients who had undergone elective hip arthroplasty surgery within 3 years prior to answering the survey. This might indicate—at least to a certain amount—some experience with hip-surgery-related issues.

In sum, this study adds to the literature by presenting results of 2 surveys among 322 patients and 187 referring physicians from the German outpatient sector to identify the most important publicly reported hospital quality information for hospital choice and to determine their relative importance. The results might support us in learning how to weight different quality measures when calculating aggregated composite measures based on consumer-based weighting. It could be shown that patients and referring physicians have similar interests with regard to the selection of publicly available quality information for the choice of hospitals. Nevertheless, we saw meaningful differences between patients and referring physicians in the relative weight of relevant information. This might have a significant impact on the hospital ranking results for each study group. Future research should address in detail how to convert hospital quality results into (numeric) scores for calculating composite measures. Besides, by understanding factors that influence patients’ hospital choice decisions, referring physicians may become more sensitive to patients’ preferences, which may have a positive effect on the process and outcomes of shared decision making.

### Supplementary Information

Below is the link to the electronic supplementary material.

Supplementary file1 (DOCX 49 KB)

## Data Availability

Data are available on request from the corresponding author (martin.emmert@uni-bayreuth.de).
