# Supplementary material for: The elicitation of patient and physician preferences for calculating consumer-based composite measures on hospital report cards: results of two discrete choice experiments
Source: Eur J Health Econ. 2023 Dec 15;25(6):1071–85. doi: 10.1007/s10198-023-01650-2 (PMC11283427; doi:10.1007/s10198-023-01650-2)
Supplement: Supplementary file 1 — Supplementary file1 (DOCX 49 KB) [file 10198_2023_1650_MOESM1_ESM.docx]

**The elicitation of patient and physician preferences for calculating consumer-based composite measures on Hospital Report Cards: Results of two discrete choice experiments**

***Supplemental material***

Supplemental material 1. Results from literature review and qualitative research steps for the patients related DCE experiment (selected measures are indicated in *italics*)

| Hospital information criteria | | Relevance of information for hospital choice | | | |
| --- | --- | --- | --- | --- | --- |
|  |  | Literature Review^#^ | Mailed survey before qualitative interviews | | Qualitative interviews^#^ |
|  |  |  | Mean | SD |  |
| 1 | Distance (home to hospital) | High | 3.05 | 1.16 | Middle |
| 2 | Ownership | High | 2.65 | 1.01 | Low |
| 3 | University hospital/teaching hospital | High | 3.45 | 1.07 | Middle |
| *4* | *The number of cases treated* | *Middle* | *4.50* | *0.74* | *High* |
| *5* | *Endocert Certificate* | *High* | *4.75* | *0.43* | *Middle* |
| 6 | Mortality rate | Middle | 3.95 | 1.28 | Low |
| *7* | *Postoperative complication rate* | *High* | *4.45* | *0.74* | *High* |
| 8 | General complication rate | High | 4.05 | 0.86 | Low |
| *9* | *Confirmed diagnosis rate** | *-* | *4.80* | *0.51* | *Middle* |
| *10* | *Prevention of falls measures** | *-* | *4.30* | *0.64* | *Middle* |
| *11* | *Mobility at hospital discharge** | *-* | *4.35* | *0.79* | *Middle* |
| 12 | Inability to walk at discharge* | - | 4.25 | 0.94 | Middle |
| 13 | 1-year revision rate | Middle | 3.90 | 1.18 | Low |
| * Those measures were added based on the German hospital quality report 2020 but were not identified by means of the literature review.  ^#^ Relevance was rated as low, middle, or high based on study results or semi-structured interviews. | | | | | |

**Supplemental material 2.** Importance of different information items for the hospital decision among patients (n = 322) and referring physicians (n = 187)
(p value was calculated using t tests)

| Information items for the hospital decision | Importance of different information items  [Mean (SD)]* | | | Most important information item^$^  [n (%)] | |
| --- | --- | --- | --- | --- | --- |
|  | Patients  (n = 299-314) | Referring physicians  (n = 187) | p | Patients  (n = 265) | Referring physicians  (n = 181) |
| Confirmed diagnosis (hip surgery) rate | 4.65 (0.80) | 3.67 (1.23) | <.001 | 118 (44.5%) | 13 (7.2%) |
| Endocert Certificate | 4.09 (1.03) | 3.05 (1.16) | <.001 | 32 (12.1%) | 9 (5.0%) |
| The number of cases treated | 4.35 (0.90) | 4.09 (0.98) | .002 | 62 (23.4%) | 65 (35.9%) |
| Complication rate | 4.54 (0.81) | 4.19 (1.06) | <.001 | 18 (6.8%) | 53 (29.3%) |
| Mobility at hospital discharge | 4.60 (0.73) | 3.31 (1.18) | <.001 | 35 (13.2%) | 4 (2.2%) |
| Prevention of falls measures | 4.29 (0.97) | n.a. | n.a. | 0 (0.0%) | n.a. |
| 1-year revision surgery rate | n.a. | 4.00 (1.21) | n.a. | n.a. | 37 (20.4%) |
| * Importance of items is rated on a 1–5 scale [1 not all important; 5 extremely important]  $ Which information was most important for your hospital choice? | | | | | |

**Supplemental material 3.** Parameters estimated from the multinomial logit (MNL) models (Discrete Choice Experiment; patients vs. physicians)

| Attributes | Patients (n = 322) | | | | Referring physicians (n = 187) | | | |
| --- | --- | --- | --- | --- | --- | --- | --- | --- |
|  | Coeff. | SE | z | p | Coeff. | SE | z | p |
| Confirmed diagnosis rate | 0.437 | 0.033 | 13.24 | <.001 | 0.390 | 0.052 | 7.54 | <.001 |
| Endocert Certificate | 0.341 | 0.033 | 10.23 | <.001 | 0.310 | 0.047 | 6.62 | <.001 |
| The number of cases treated | 0.547 | 0.036 | 15.07 | <.001 | 0.696 | 0.051 | 13.61 | <.001 |
| Complication rate | 0.560 | 0.037 | 15.08 | <.001 | 0.546 | 0.050 | 10.83 | <.001 |
| Mobility at hospital discharge | 0.551 | 0.036 | 15.15 | <.001 | 0.236 | 0.046 | 5.14 | <.001 |
| Prevention of falls measures | 0.431 | 0.035 | 12.35 | <.001 | n.a. | n.a. | n.a. | n.a. |
| 1-year revision surgery rate | n.a. | n.a. | n.a. | n.a. | 1.014 | 0.053 | 19.10 | <.001 |
| *Model constant (Intercept)* | 0.325 | -0.045 | -7.29 | <.001 | n.a. | n.a. | n.a. | n.a. |
